# Lung Mean Dose Prediction in Transarterial Radioembolization (TARE): Superiority of [^166^Ho]-Scout Over [^99m^Tc]MAA in a Prospective Cohort Study

**DOI:** 10.1007/s00270-023-03656-y

**Published:** 2024-02-07

**Authors:** Martijn E. H. M. Wagemans, Arthur J. A. T. Braat, Rob van Rooij, Maarten L. J. Smits, Rutger C. G. Bruijnen, Jip F. Prince, Guus M. Bol, Hugo W. A. M. de Jong, Marnix G. E. H. Lam

**Affiliations:** 1 Department of Radiology and Nuclear Medicine, University Medical Center Utrecht, P.O. Box 85500, 3508 GA Utrecht, The Netherlands; 2 Department of Medical Oncology, University Medical Center Utrecht, Utrecht, The Netherlands

**Keywords:** Radioembolization, Holmium-166, SPECT/CT, Lung mean dose, Radiation pneumonitis

## Abstract

**Purpose:**

Radiation pneumonitis is a serious complication of radioembolization. In holmium-166 ([^166^Ho]) radioembolization, the lung mean dose (LMD) can be estimated (eLMD) using a scout dose with either technetium-99 m-macroaggregated albumin ([^99m^Tc]MAA) or [^166^Ho]-microspheres. The accuracy of eLMD based on [^99m^Tc]MAA (eLMD_MAA_) was compared to eLMD based on [^166^Ho]-scout dose (eLMD_Ho-scout_) in two prospective clinical studies.

**Materials and Methods:**

Patients were included if they received both scout doses ([^99m^Tc]MAA and [^166^Ho]-scout), had a posttreatment [^166^Ho]-SPECT/CT (gold standard) and were scanned on the same hybrid SPECT/CT system. The correlation between eLMD_MAA_/eLMD_Ho-scout_ and LMD_Ho-treatment_ was assessed by Spearman’s rank correlation coefficient (*r*). Wilcoxon signed rank test was used to analyze paired data.

**Results:**

Thirty-seven patients with unresectable liver metastases were included. During follow-up, none developed symptoms of radiation pneumonitis. Median eLMD_MAA_ (1.53 Gy, range 0.09–21.33 Gy) was significantly higher than median LMD_Ho-treatment_ (0.00 Gy, range 0.00–1.20 Gy; *p* < 0.01). Median eLMD_Ho-scout_ (median 0.00 Gy, range 0.00–1.21 Gy) was not significantly different compared to LMD_Ho-treatment_ (*p* > 0.05). In all cases, eLMD_MAA_ was higher than LMD_Ho-treatment_ (*p* < 0.01). While a significant correlation was found between eLMD_Ho-scout_ and LMD_Ho-treatment_ (*r* = 0.43, *p* < 0.01), there was no correlation between eLMD_MAA_ and LMD_Ho-treatment_ (*r* = 0.02, *p* = 0.90).

**Conclusion:**

[^166^Ho]-scout dose is superior in predicting LMD over [^99m^Tc]MAA, in [^166^Ho]-radioembolization. Consequently, [^166^Ho]-scout may limit unnecessary patient exclusions and avoid unnecessary therapeutic activity reductions in patients eligible for radioembolization.

*Trail registration*: NCT01031784, registered December 2009. NCT01612325, registered June 2012.

**Graphical Abstract:**

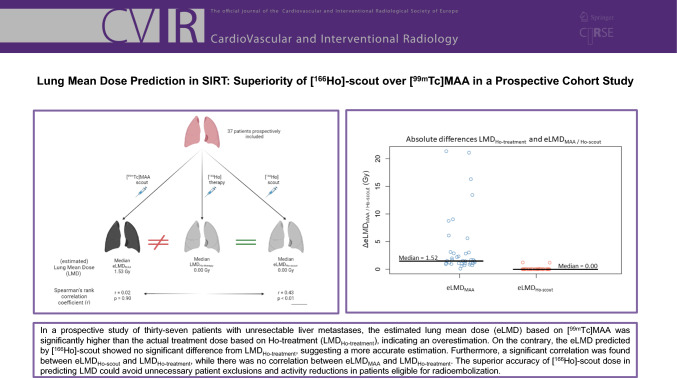

## Introduction

During hepatic radioembolization, microspheres with beta-emitting isotopes of either yttrium-90 (^90^Y) or holmium-166 ([^166^Ho]) are injected via catheterization of the hepatic artery [1]. Treatment is preceded by injection of a scout dose to simulate distribution, most commonly using technetium-99 m-macroaggregated albumin ([^99m^Tc]MAA) [2]. The purpose of this scout procedure is threefold: (1) to analyze the anticipated intrahepatic distribution of activity after treatment; (2) to exclude unacceptable extrahepatic abdominal activity caused by hepato-gastro-intestinal collaterals; (3) to estimate the anticipated radiation absorbed dose in the lungs caused by shunting. The latter is of importance to avoid radiation pneumonitis, a rare but serious complication.

Arteriovenous anastomoses in liver parenchyma or in tumors allow for shunting of particles and may cause depositions of activity in the lungs. This can severely affect respiratory function [2]. Radiation pneumonitis typically occurs 1–6 months posttreatment and is clinically characterized by dry cough and progressive exertional dyspnea, potentially becoming life-threatening [3].

Patients are generally excluded from radioembolization if predicted lung mean dose (LMD) exceeds 30 Gy for a single treatment and/or 50 Gy for multiple treatments [1]. In a survey among radioembolization centers, 48% of respondents answered that up to a quarter of their patients were considered ineligible for therapy, based on lung shunting as assessed with [^99m^Tc]MAA [4]. While this finding highlights the substantial impact of lung shunting on clinical practice, there is scientific evidence suggesting that lung shunting is largely overestimated by [^99m^Tc]MAA, especially when using planar imaging [2–5]. Several explanations for the poor predictive value of [^99m^Tc]MAA have been identified, including inaccurate quantification of [^99m^Tc]MAA, particle size reduction by fragmentation of the albumin aggregates and differences in biodistribution of [^99m^Tc]MAA compared to the treatment particle [2–6].

Treatment with [^166^Ho]-microspheres can be preceded by a scout dose consisting of the same microspheres, instead of [^99m^Tc]MAA. For [^166^Ho], beta-decay is accompanied by the emission of gamma photons (81 keV, 6.2% abundance), enabling the use of quantitative SPECT/CT to predict distribution of [^166^Ho]-microspheres [2]. Braat et al. showed that use of [^166^Ho]-scout dose is safe, even if significant extrahepatic depositions occur [7].

Previously, Elschot et al. compared the performance of [^99m^Tc]MAA and [^166^Ho]-scout for estimation of LMD prior to [^166^Ho]-radioembolization in 14 patients with unresectable liver metastases [2]. In that clinical phase I study, [^166^Ho]-scout proved to be more accurate than [^99m^Tc]MAA in predicting LMD, with [^99m^Tc]MAA significantly overestimating LMD compared to posttreatment [^166^Ho]-SPECT/CT [2]. Although significant, differences were only validated in a limited number of patients. In the present study, the clinical value of [^166^Ho]-scout versus [^99m^Tc]MAA-scout for LMD prediction was investigated in an expanded patient population, consisting of both the initial phase I study and a subsequent phase II within-patient comparison study.

## Materials and Methods

### Patients

All patients from the prospective phase I and II Holmium Embolization Particles for Arterial Radiotherapy (HEPAR) studies were included (Clinicaltrials.gov numbers NCT01031784 and NCT01612325) [7, 8]. Each patient had unresectable liver metastases treated with [^166^Ho]-microspheres. The institutional review board approved the studies and all patients provided written informed consent before enrollment [6]. Patients were included in the present analysis if they received both scout doses ([^99m^Tc]MAA and [^166^Ho]-scout), had a posttreatment [^166^Ho]-SPECT/CT (defined as gold standard) and were all scanned on the same hybrid SPECT/CT system.

Between December 2009 and March 2015, 53 patients were included in the phase I and II HEPAR studies. All patients received [^99m^Tc]MAA, [^166^Ho]-scout and subsequent [^166^Ho]-treatment dose. Of these, sixteen patients were excluded from the analysis due to scanning on a non-hybrid SPECT system (10 patients) or unavailability of a posttreatment scan (6 patients), resulting in a total of 37 patients for analysis. The majority of patients presented with colorectal carcinoma (19/37, 51.4%) (Table 1).

**Table 1:** Patient characteristics and details of treatment

Patient characteristics	
Number of patients	37
Gender (%)	
Male	21 (56.8)
Female	16 (43.2)
Age (years) median (range)	64 (40–87)
Primary tumor type: number (%)	
Colorectal carcinoma	19 (51.4)
Uveal melanoma	4 (10.8)
Cholangiocarcinoma	5 (13.5)
Breast carcinoma	4 (10.8)
Neuroendocrine tumor	2 (5.5)
Gastric carcinoma	1 (2.7)
Thymoma	1 (2.7)
Pancreatic carcinoma	1 (2.7)
Details of treatment	
Median interval between [^99m^Tc]MAA–[^166^Ho] (days) (range)	7 (2–20)
Treated liver volume (mL) median (range)	1757 (76–3509)
Diameter largest tumor (mm) median (range)	56 (18–158)
Net injected activity (MBq) median (range)	
Pretreatment [^99m^Tc]MAA:	142 (65–491)
Pretreatment [^166^Ho]-microspheres:	261 (147–292)
Treatment [^166^Ho]-microspheres:	6159 (2207–12897)
Treated liver lobes	
Bilobar*	30
Right lobar	6
Left lobar	1

*All bilobar treatments were performed in a single session

### Procedure

Several days before treatment a preparatory angiography was performed. An aimed total activity of 150 MBq [^99m^Tc]MAA (0.8 mg, approximately 1.8 million particles, TechneScan LyoMAA; Mallinckrodt Medical B.V., Petten, The Netherlands) was injected at one or more injection positions, followed by SPECT/CT [6]. The median injected activity was 142 MBq, range 65–491 MBq. To avoid degradation of [^99m^Tc]MAA, activity was prepared on demand, immediately before use and imaging was performed immediately after angiography. On the day of treatment, exact injection positions were reproduced, and patients first received an aimed scout dose of 250 MBq [^166^Ho]-microspheres in the morning. The scout dose consisted of approximately 60 mg; 3 million microspheres, with a median injected activity of 261 MBq (range 147–292 MBq). A vascular sheath was left in the common femoral artery to facilitate repeat catheterization in the afternoon. If subsequent SPECT/CT revealed no contra-indications for radioembolization, catheterization was repeated and the [^166^Ho]-microspheres treatment dose was administrated in the afternoon. The [^166^Ho]-microspheres were produced on site (University Medical Center Utrecht, Utrecht, the Netherlands) [9, 10]. Median administered treatment activity of [^166^Ho]-microspheres per procedure was 6.159 MBq (range 2.207–12.897 MBq). In all patients, the injection positions in the three procedures were assessed as being adequately matched. Provided that catheters were situated within the same vessel, any positional variance was considered inconsequential to the magnitude of the lung shunt. In the majority of treatments (25/37, 67.6%), injections were performed sequentially in the left and right hepatic artery. Follow-up consisted of physical examinations, blood work and imaging during a period of at least 3 months after [^166^Ho]-treatment [8]. Adverse events were scored according to the Common Toxicity Criteria for Adverse Events version 3.0 [8].

### Imaging

All SPECT/CT images were acquired on the same dual headed SPECT/CT camera (Symbia T16, Siemens Health Care). [^99m^Tc]MAA-SPECT images were acquired using a low energy collimator, 128 × 128 matrix, 120 angles (20 s. per projection) over a noncircular 360° orbit and a 140-keV ± 7.5% photopeak energy window. [^166^Ho]-SPECT data were acquired using a medium energy collimator, 128 × 128 matrix with 120 angles over a noncircular 360° orbit and a 81-keV ± 7.5% photopeak window. Low-dose CT data were acquired and used to create a CT-derived attenuation map (Syngo MI Applications; Siemens Healthcare). All SPECT/CT images enclosed the entire liver and the basal lung fields. [^99m^Tc]MAA and [^166^Ho]-SPECT were reconstructed using clinical reconstructions, applying previous protocols [2].

### Quantitative Analysis

Using SPECT/CT images, volumes of interest (VOIs) were segmented on corresponding co-registered abdominal low-dose CT scans, using ITK-snap (version 3.8.0) [11]. The liver VOI was manually delineated. To minimize intraobserver differences, the lungs were automatically delineated using a freely available pre-trained convolutional neural network, lung mask, using a U-net model (R231) [12]. The body contour was obtained by threshold-based segmentation of the low-dose CT in order to obtain total body counts in the co-registered SPECT image. All images were visually checked to ensure correct segmentation and registration. Erroneous registration of liver activity in lungs was expected, due to co-registration errors, partial volume effect and/or patient breathing. Therefore, a 3D 2 cm margin was automatically added around the liver VOI. The voxels in the 3D liver + 2 cm were excluded from the lung VOI [2].

To maximize accuracy, estimated LMD (eLMD) was based on measured activity in the left lung alone, as it was less prone to erroneous registration of liver activity in the lung VOI [13]. The LMD was assumed to be equal in both lungs. The eLMD on all SPECT/CT’s was calculated using the following formula,

$${\text{eLMD}}\left({\text{Gy}}\right)=\frac{\mathrm{Counts\,left\,lung\,VOI}}{\mathrm{Counts\,total\,body}}*\frac{{{\text{A}}}_{{\text{net}}}\left({\text{GBq}}\right) * 15.87 (\frac{{\text{J}}}{{\text{GBq}}})}{{{\text{M}}}_{\mathrm{left\,lung\,VOI}}({\text{kg}})}$$

in which A_net_ is the net administered activity (calibrated activity for [^166^Ho]-microspheres treatment–measured residual activity in the administration system after [^166^Ho]-microspheres treatment), 15.87 J/GBq the conversion factor of energy deposition and M_left lung VOI_ the calculated mass of the left lung VOI (volume left lung VOI multiplied by an assumed lung density of 0.3 g/mL) [2].

### Statistical Analysis

The LMD_Ho-treatment_ was assessed by posttreatment [^166^Ho]-SPECT/CT (i.e., the gold standard). The correlation between eLMD_MAA_/eLMD_Ho-scout_ and LMD_Ho-treatment_ was assessed by calculating the Spearman’s rank correlation coefficient (*r*). Absolute differences in eLMD_MAA/Ho-scout_ minus LMD_Ho-treatment_ (ΔeLMD_MAA/Ho-scout_) were calculated to compare the predictive value of both methods. Bland–Altman analyses to assess the correlation between eLMD_MAA_/eLMD_Ho-scout_ and LMD_Ho-treatment_ were not conducted, given the median LMD_Ho-treatment_ was (near) zero (see results section), indicating that differences were explained by observed lung shunting during scout procedures. Descriptive parameters are presented as medians and range. Statistical data analysis was performed using a commercial statistical software package (SPSS for Windows, version 21.0; SPSS Inc.). Wilcoxon signed rank test was used to analyze paired data (significance level 0.05), since normal distribution could not be assumed.

## Results

**Fig. 1:**
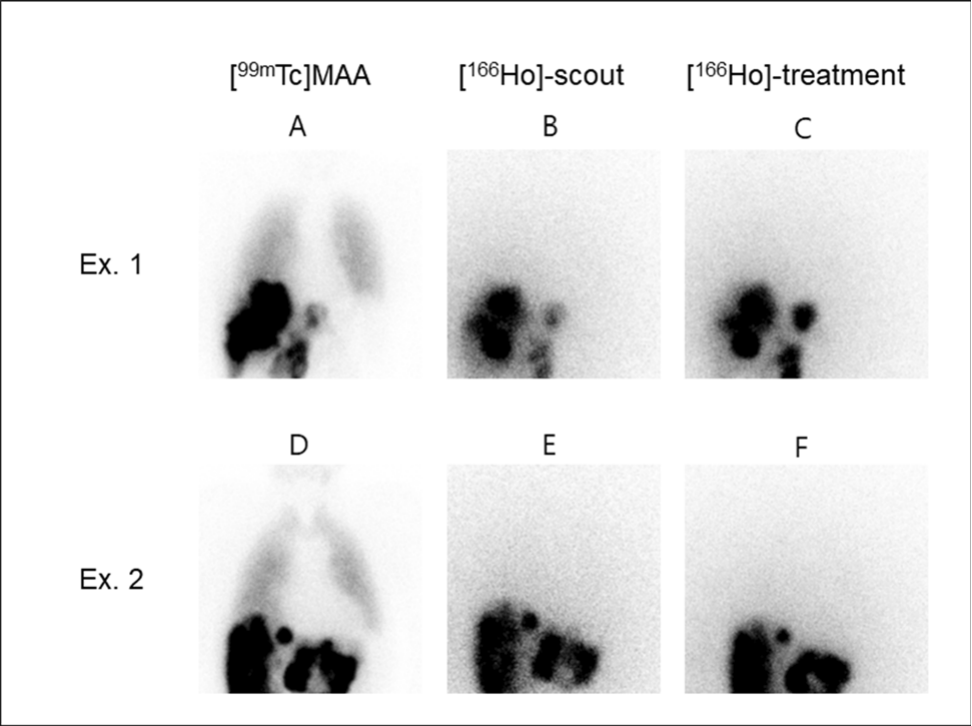
Planar images of two patients illustrating the difference in activity distribution. The eLMD_MAA_ for the first patient was 21.3 Gy, while both eLMD_Ho-scout_ and LMD_Ho-treatment_ were 0.0 Gy (**A**–**C**). The second patient had an eLMD_MAA_ of 21.1 Gy, with the eLMD_Ho-scout_ and LMD_Ho-treatment_ both measured as 0.0 Gy (**D**–**F**). From left to right; (A/D) pretreatment [^99m^Tc]MAA scintigraphy, (B/E) pretreatment [^166^Ho]-scout scintigraphy and (C/F) posttreatment [^166^Ho]-scintigraphy

Median follow-up was 4 months (range 1–14 months). During follow-up, none of the included patients showed symptoms of radiation pneumonitis. Median LMD_Ho-treatment_ was 0.00 Gy (range 0.00–1.20 Gy). eLMD_MAA_ was significantly higher with a median of 1.53 Gy (range 0.09–21.33 Gy) (*p* < 0.01). The eLMD_Ho-scout_ was not significantly different from LMD_Ho-treatment_ (median 0.00 Gy, range 0.00–1.21 Gy) (*p* > 0.05) (Fig. 1).

**Fig. 2:**
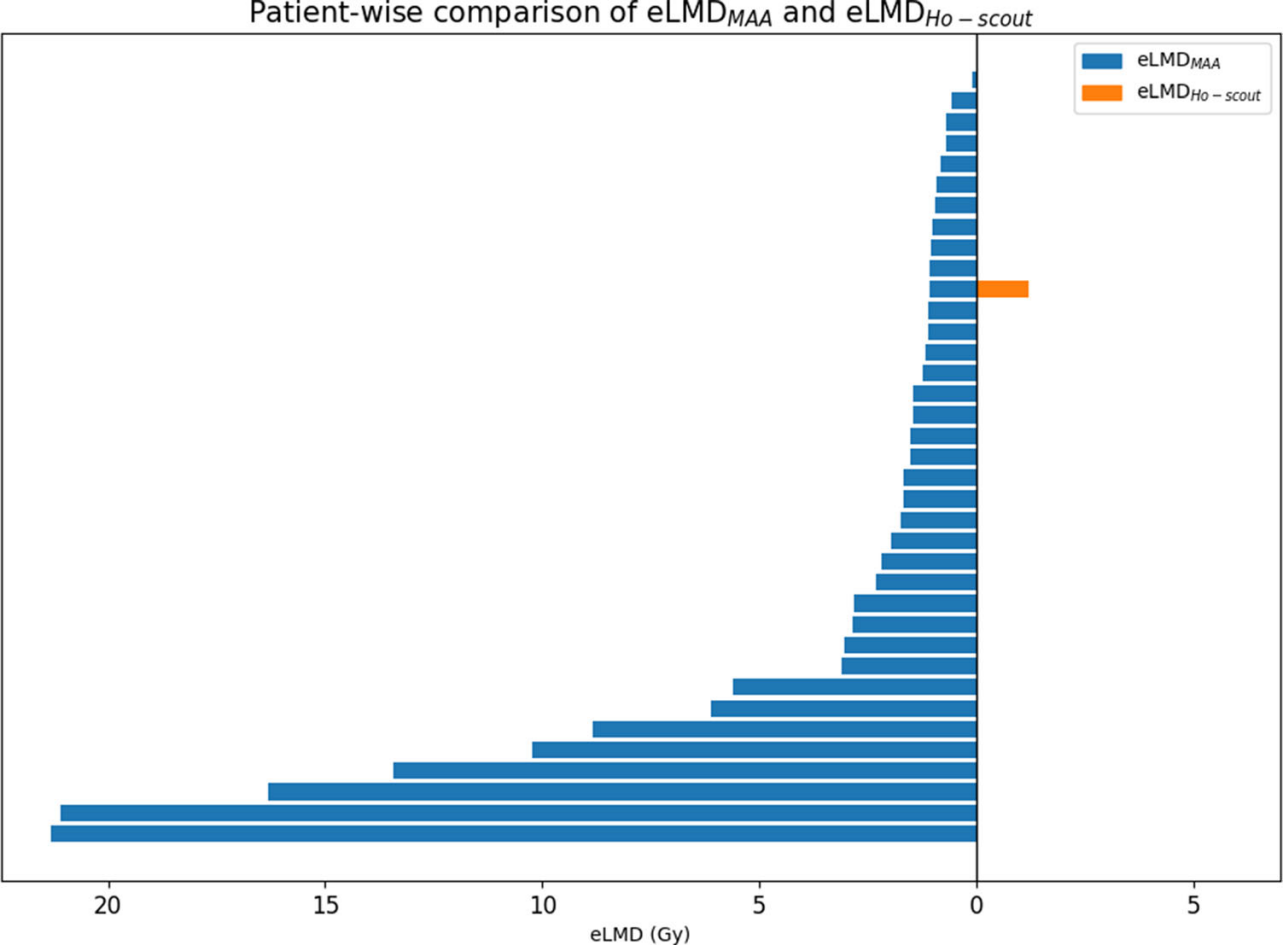
Diverging bar chart showing the estimated lung mean dose (eLMD) per subject for [^99m^Tc]MAA-scout (blue) and the [^166^Ho]-scout (orange).The eLMD_Ho-scout_ bars may not be visible, due to their relatively low values

**Fig. 3:**
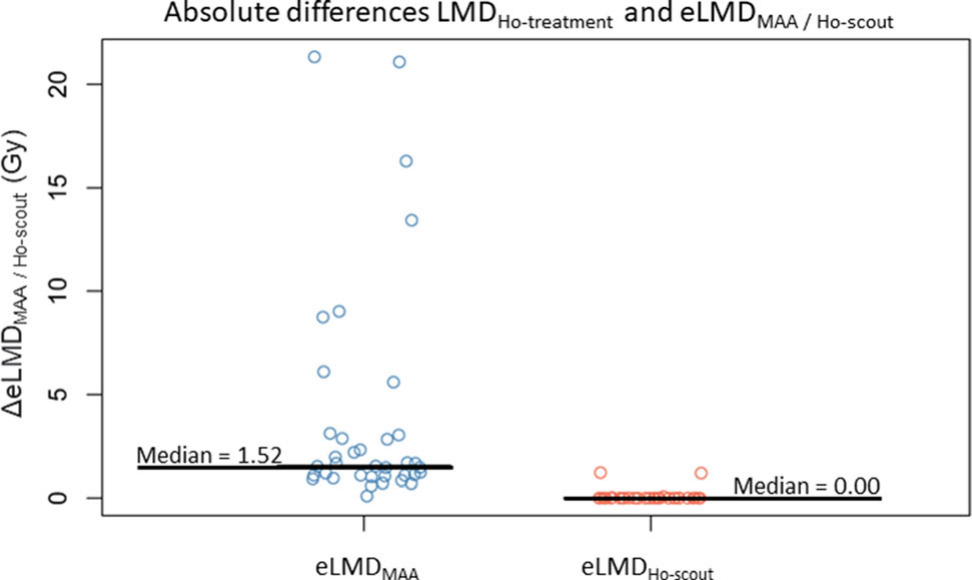
Scatterplot of absolute differences between eLMD_MAA_ and LMD_Ho-treatment_ (ΔeLMD_MAA_), and eLMD_Ho-scout_ and LMD_Ho-treatment_ (ΔeLMD_Ho-scout_). The median values are represented by the black lines

In all cases, eLMD_MAA_ was higher than the LMD_Ho-treatment_ (Fig. 2). While a significant, positive correlation was found between eLMD_Ho-scout_ and LMD_Ho-treatment_ (*r* = 0.43, *p* < 0.01), there was no correlation between eLMD_MAA_ and LMD_Ho-treatment_ (*r* = 0.02, *p* = 0.90). The median ΔeLMD_MAA_ of 1.52 Gy (range 0.09–21.33 Gy) was significantly higher than median ΔeLMD_Ho-scout_ of 0.00 Gy (range 0.00–1.21 Gy) (*p* < 0.01) (Fig. 3).

Regarding the ΔeLMD_MAA_, eight out of 37 patients (21.6%) demonstrated a difference greater than 5 Gy. Two out of 37 (5.4%) showed an absolute difference exceeding 20 Gy. These two patients were diagnosed and treated for colorectal carcinoma and neuroendocrine tumor liver metastases, respectively. Interestingly, intrahepatic cholangiocarcinoma (ICC) patients constituted half of the cases with differences exceeding 5 Gy. Among the five ICC patients included in this study, four out of five (80%) displayed a difference greater than 5 Gy. The median eLMD_Ho-scout_ for ICC patients was 0.0 Gy (range 0.00–0.00 Gy), while the median eLMD_MAA_ was 6.11 Gy (range 0.09–16.3 Gy). Median time interval between [^99m^Tc]MAA and [^166^Ho]-scout was seven days (range 2–20 days). No (serious) adverse events possibly, probably or definitively related to the [^166^Ho]-scout were registered.

## Discussion

The lung absorbed dose based on posttreatment [^166^Ho]-SPECT/CT and estimated by [^166^Ho]-scout were both significantly lower than estimations based on [^99m^Tc]MAA. None of the patients developed signs of radiation pneumonitis.

As highlighted by van Elschot et al., the differences between [^99m^Tc]MAA and [^166^Ho]-scout are primarily attributed to the distinct particle characteristics and biodistribution patterns of [^99m^Tc]MAA and [^166^Ho]-microspheres [2].

The higher accuracy of [^166^Ho]-scout for LMD prediction confirms previous phase I findings by Elschot et al. [2]. The methods used in the current study and the phase I study by Elschot et al. differed slightly. The eLMD calculated by Elschot et al. was based on the registered activity in both lungs. In the current study, the right lung was excluded to minimize erroneous capture of liver activity. As the lung perfusion between the right and left lung was assumed to be nearly symmetrical, the left lung was considered representative for eLMD [14].

The [^99m^Tc]MAA dose deposition in the lungs observed in this study, 1.53 Gy (range 0.09–21.33 Gy), is in line with prior reports. A study on predictive lung dosimetry in ^90^Y-radioembolization, using [^99m^Tc]MAA-SPECT/CT, reported a median eLMD_MAA_ of 4.51 (range 0.85–18.87) [15]. Recently, Stella et al. investigated the occurrence of radiation pneumonitis after ^90^Y-radioembolization in relation to LMD. The eLMD was calculated on [^99m^Tc]MAA planar scintigraphy by multiplying LSF with administered therapeutic activity. The actual LMD was determined on posttreatment ^90^Y-PET. In line with this study, a median eLMD_MAA_ of 3.5 Gy (range 0.2–89.0 Gy) and an actual median LMD of 1 Gy (range 0.0–22.1 Gy) were reported.[5] However, eLMD_MAA_ derived from planar scintigraphy is known to overestimate LMD compared to SPECT/CT measurements [3, 16].

Likewise, in the context of resin [^90^Y] radioembolization, the potential advantages of using the same particle for scout and treatment have been investigated. In a single-arm clinical trial, involving 30 patients with HCC, the efficacy and safety of 0.56 GBq resin [^90^Y] microspheres (scout^90^Y) were compared with [^99m^Tc]MAA for predicting the therapeutic resin [^90^Y] dose [17]. The mapping procedures using both [^99m^Tc]MAA and scout^90^Y were performed on the same day, with treatment activity administered after three days. Scout^90^Y, using attenuation corrected SPECT/CT images, outperformed [^99m^Tc]MAA SPECT/CT in predicting lung shunt fraction (LSF). In the case of LSF, scout^90^Y demonstrated a strong linear correlation with the therapeutic dose (*r* = 0.76, *p* < 0.001), in contrast to [^99m^Tc]MAA’s weak correlation (*r* = 0.39, *p* = 0.032). These findings underscore the potential advantages of using a surrogate scout over [^99m^Tc]MAA for LMD prediction in glass [^90^Y] radioembolization as well.

Accurate eLMD is important, not only to prevent radiation pneumonitis, but even more to avoid unnecessary dose reduction and/or patient exclusion [6]. LMD predictions are typically made by quantification of [^99m^Tc]MAA distribution on planar scintigraphy [3]. The LSF is determined by dividing the counts in the lung area by the total counts in both the lung and liver regions [5]. The resulting LSF may then be multiplied by the planned therapeutic activity to acquire an eLMD. For all commercially available radioembolization particles, the upper dose limit to the lungs is set at 30 Gy for single radioembolization treatment [1, 18]. To date, this is also the case for [^166^Ho]-microspheres; however, the rationale for this maximum is based on limited research and adopted from ^90^Y data. Moreover, in the above-mentioned study by Stella et al., only two out of 14 patients with an eLMD_MAA_ above 30 Gy developed radiation pneumonitis after ^90^Y-treatment [5]. These results suggest that treatment adjustments or exclusion based on eLMD_MAA_ seem to be unjustified in numerous cases. In the prospective SARAH- and EPOCH-trial, 6.2% (14/226) and 1.8% (4/215) of patients, respectively, were excluded based on eLMD by planar [^99m^Tc]MAA imaging. This stresses the need for a more accurate prediction method for LMD. At the same time, the 30 Gy eLMD threshold will be difficult to validate as the number of reported radiation pneumonitis cases in clinic is very low (< 1%) [5].

In line with a previous report by our group, no (serious) adverse events related to [^166^Ho]-scout were registered during follow-up [7]. Moreover, in the recently completed SIM and HEPAR PLuS studies, [^166^Ho]-scout was used instead of [^99m^Tc]MAA, further confirming its safety [19, 20].

Regarding the quantification method, the used lung dosimetry model was based on commonly applied assumptions, including minimal lung absorbed dose from extra-pneumonic tissue, complete local energy absorption and similar lung density for all patients. This impacts the accuracy of the LMD calculations, since lung density depends on the presence of lung pathologies, scanning position and inclusion of lung vasculature [13, 18]. Since the same model was applied for [^99m^Tc]MAA and [^166^Ho]-scout analysis, the expected effect of these factors on the comparison was also limited.

Lastly, patterns of vascularization differ per tumor type. Our study was primarily based on metastatic colorectal tumors (51.4%). More hypervascular tumor types, such as hepatocellular carcinoma (HCC), are more susceptible to arteriovenous shunting, which consequently leads to a higher LMD [18, 21, 22]. HCC patients were not part of the present study. However, five patients with ICC, another hypervascular tumor, were included. The LMD was overestimated in four out of five ICC patients when using [^99m^Tc]MAA, while the eLMD from [^166^Ho]-scout was in line with the actual LMD. It is therefore likely that [^166^Ho]-scout superiority in estimating LMD will hold in hypervascular tumors due to its inherent physical benefits over [^99m^Tc]MAA. With the increase in use of [^166^Ho]-scout dose, it is expected that definitive data in hypervascular tumors, including HCC, will become available within the coming years. This study has several limitations regarding the administration technique used. First, the [^99m^Tc]MAA-scout procedure and [^166^Ho]-treatment were performed on different days, while [^166^Ho]-scout and [^166^Ho]-treatment were performed on the same day. Second, [^99m^Tc]MAA and [^166^Ho]-scout administration methods were different, bolus syringe injection for [^99m^Tc]MAA versus a dedicated administration box for both [^166^Ho]-scout and [^166^Ho]-treatment. The administration pressure, volume and velocity may influence intravascular flow dynamics of the particles and thus particle distribution.[23] Third, even slight differences in injection position may lead to different flow dynamics for [^99m^Tc]MAA and [^166^Ho]-scout. Fortunately, these factors are less likely to influence the assessment of lung shunting compared to the known influence on intrahepatic distribution [6]. Other limitations relate to the imaging techniques used. Due to its narrow field of view, SPECT imaging did not always include the upper lung regions. This limited the accuracy of LMD estimation to a certain extent since quantification depended on a specific area of the left lung only. Even though commonly assumed in the literature, distribution of microspheres in the lungs is not homogenous. Gravitational dependence of alveolar and vascular pressure results in preferential perfusion of the lower dorsal lung regions compared to the apex [24]. Nevertheless, missing upper regions on SPECT/CT images are expected to have a small effect on the current comparison. Furthermore, the emission spectrum of [^166^Ho] is not ideal for SPECT imaging, due to the high-energy gamma emissions which cause a significant down-scatter contribution in the 80.6 keV photopeak window. Accurate scatter correction methods relying on Monte Carlo simulations are often not available in clinical practice. Using conventional energy-window-based scatter correction, low count regions are more prone to inaccurate quantification due to under- or over correction.

## Conclusion

[^166^Ho]-scout is superior in predicting lung mean dose over [^99m^Tc]MAA. Using [^166^Ho]-scout may avoid unnecessary patient exclusions and therapeutic activity reductions in patients eligible for radioembolization.
